# Functional improvement after Total Knee Arthroplasty Revision: New observations on the dimensional nature of outcome

**DOI:** 10.1186/1749-799X-2-25

**Published:** 2007-12-07

**Authors:** Kevin J Mulhall, Hassan M Ghomrawi, Boris Bershadsky, Khaled J Saleh

**Affiliations:** 1 Department of Orthopaedic Surgery, Mater Misericordiae University Hospital, Dublin, Ireland; 2 Clinical Outcome Research Center, University of Minnesota, Minneapolis, MN 55455, USA; 3 Department of Orthopaedic Surgery, University of Virginia, Charlottesville, VA 22908, USA

## Abstract

**Background:**

Despite the numerous outcomes measures described it remains unclear what aspects of patient outcome are important in determining actual improvement following total knee arthroplasty revisions (TKAR). We performed a prospective cohort study of TKAR to determine the components of clinical improvement and how they are related and best measured.

**Methods:**

An improvement scale was devised utilizing data from 186 consecutive TKAR patients on SF-36 physical (PCS) and mental (MCS) components, Western Ontario and McMaster Universities Osteoarthritis (WOMAC) Index, Knee Society Score (KSS), a novel Activity Scale (AS) and a physician derived severity assessment scale performed both preoperatively and at 6 month post-operative follow-up. The change in each of these scores was analyzed using factor analysis, deriving a composite improvement scale.

**Results:**

All the instruments demonstrated statistically significantly better scores following TKAR (except the SF-36 MCS). Furthermore, all significant correlations between the scores were positive. Statistical factor analysis demonstrated that scores could be arranged into 4 related factor groupings with high internal consistency (Cronbach Alpha = 0.7). Factor 1 reflected patient perceived functional outcomes, Factor 2 activity levels, Factor 3 the MCS and Factor 4 the KSS.

**Conclusion:**

This study demonstrates that improvement following TKAR has a multidimensional structure. The improvement scales represent a more coordinated method of the previously fragmented analysis of TKAR outcomes. This will improve assessment of the actual effectiveness of TKAR for patients and what aspects of improvement are most critical.

## Background

The concept of improvement following arthroplasty surgery is multidimensional, with outcome results varying, both in meaning and in quantity, depending on the reporter – patient or surgeon – and on the dimension under evaluation (e.g., pain, function, range of motion, etc.). There is currently a lack in the literature, however, of any real attempt to investigate the relationships between the different outcomes measures and furthermore to analyze this multidimensional nature of improvement following surgical intervention [1].

The various instruments currently used, both disease specific and general health measures, assess outcomes and are potentially important in guiding future practice by demonstrating the effects of therapies in particular patients at particular times [2-4]. However, the scope and number of instruments can be confusing. The aim of all of them is clear: trying to demonstrate and measure patients' improvement accurately. Accuracy here allows us to compare results between different groups of interventions and different patients and also to predict outcomes and thus apply relevant interventions. So, although patient improvement is generally perceived to be the sine qua non of any surgical intervention, uncertainty arises when we attempt to determine exactly what tests are truly relevant and independent, and, more fundamentally, what constitutes actual improvement.

The very existence of this array of tests and instruments indicates that improvement is a multi-dimensional entity that is probably not fully captured by any one currently available instrument. No previously described instrument or report describes or addresses this dimensional structure of improvement. The commonly used tests have been developed in a cross sectional manner at a certain point in time and then applied longitudinally [5,6]. All the commonly used instruments assess relatively important aspects of patient outcomes, for example pain, activity, function, general health, mental health, stiffness or range of movement [7-10]. It is, nonetheless, not clear that these various tests are measuring entirely independent facets of improvement or whether there is significant overlap or redundancy in their measurements from a global perspective.

The objective of the current study was thus to analyze nine of the most commonly utilized outcome measures in assessing total knee arthroplasty in a cohesive manner by factor analysis in order to determine whether they measure separate or complementary aspects of improvement. An extension of this objective was then to determine whether it is possible to categorize or describe improvement in a more streamlined and potentially useful fashion.

## Methods

A consecutive series of patients in need of a revision procedure for a failed total knee arthroplasty were prospectively followed in a multi-center cohort study involving 14 centers in the United States and one in Canada. Patients were spread relatively equally between sites and a total of 6 patients were lost to follow-up. These 6 all came from separate units. All patients had to meet specific inclusion and exclusion criteria prior to enrollment in the study. The inclusion criteria were that at the least, the tibial and/or the femoral component required reconstruction, signed informed consent was obtained from the subject, the patient was over 18 years of age, the patient was cognitively intact, fluent in English, and capable of completing the self-administered questionnaires and adhering to the study protocol, the patient had a primary TKA that had failed, not a re-revision. The exclusion criteria were patients having a TKA re-revision, revision for failed unicondylar prosthesis, patients with metastatic or primary tumour of the knee, reflex sympathetic dystrophy of the leg, subject medically unfit to undergo TKAR, progressive muscular condition (with quadriceps weakness), neurologic deficit of affected limb, knee pain associated with spinal pathology, patient declined participation.

After obtaining IRB approval from each site, patients with failed total knee arthroplasties were approached about study participation. Once the patient agreed to participate, the investigator obtained subject consent, and the subject was then included in the study. Subjects and investigators then completed respective baseline forms.

As is necessary with any multi-center study of this nature, great care was taken a priori in the design of the study to ensure uniformity of indications, data management and follow-up between centers [11]. All documentation was performed using a standard set of proforma questionnaires, for both surgeons and patients, structured so as to not permit of any deviation in data collection. Strict inclusion and exclusion criteria were applied from the outset and the coordinators that helped collect the data were blinded to study design and hypotheses.

The specific information gathered from the patients and investigators were Short Form-36 (SF-36) both mental (MCS) and physical components (PCS), the Western Ontario and McMaster Universities Osteoarthritis (WOMAC) Index (pain, stiffness, and difficulty of function), the Knee Society Score (KSS) both functional and clinical components, the Lower Extremity Activity Scale (LEAS), and a physician derived severity score, which is a visual analogue scale. Nine scales in all were thus involved in subsequent calculations. These instruments include the most commonly used in arthroplasty studies, for both primary and revision procedures. Although it might be argued that a TKAR population is potentially more heterogeneous than a primary population, these instruments are used in identical fashion for both populations and any new approach based on these instruments has to be robust enough to measure improvement in any arthroplasty cohort.

Among the less familiar scales used here, the physician derived severity score has been previously utilized and validated by the authors as an investigative tool in assessing the subjective physician judgment of the severity of the patient's condition and likelihood of good outcome, specifically as this relates to the failed or failing knee implant [11]. The LEAS is a simple, patient administered instrument developed and comprehensively validated by the current authors that assesses the actual activity level of lower limb arthritis and arthroplasty patients [12].

Baseline forms were completed prior to the revision procedure and a further set of follow-up forms were subsequently completed at six months postoperatively. As we were testing here only a new methodological approach to analyzing postoperative improvement, we did not pursue longer clinical follow up of this cohort for the purpose of this study. Each of the constituent scales used results in a single 'outcome score' for that scale. Although these scores often present difficulties in clinical interpretation for individual patients, particularly those with 'mixed' outcomes (such as good in one measurement in the scale but poor in another) they are very useful in analyzing cohort populations, and represent the best means we currently have for outcome analyses. The changes in these scores from baseline to follow up were then converted into measures of improvement by assigning a positive sign to improvement in each patient's condition. This modification was necessary as, for example, a decrease in one system might signal improvement versus another system where increasing scores indicate improvement and so on. The resultant scores for the patients were then combined in order to determine the improvement or otherwise that occurred for each system (WOMAC, KSS, LEAS, physician derived score and SF-36). Improvement for each of the scales was then normalized by its respective standard deviations so that it was possible to compare the magnitude of improvement of individual scales.

Exploratory orthogonal factor analysis with varimax rotation was applied to the change in scores between the two time points for all instruments. The essential purpose of the factor analysis was to determine if the measures of change could be grouped into factors in order to more parsimoniously describe the concept of improvement. This orthogonality or 'independence' of the factors was an assumption we made a priori, but it should be noted, however, that the dimensions of improvement are not necessarily independent. This assumption was nevertheless necessary in practice, as the use of non-orthogonal factor analysis at this point of the study would provide too much uncertainty in the factorial model. For example, changes between scores in individual patients have smaller systematic variations than single scores and because they are based on the difference between 2 scores have a higher random error than any one of the basic measures.

As the result of this orthogonal rotation, then, we obtained several factors. Each of them was represented as a combination of all nine measures of improvement. We then applied non-linear transformation of the formulas (V1 = (D1+D5+D6+D7)/4, V2 = (D3+D4+D8)/3, V3 = D2, V4 = D9) – all coefficients that were greater than 0.6 were assumed to be equal and all coefficients that were smaller than 0.6 were replaced with zero. In this equation, D1-D9 values refer to the changes of scale scores from baseline to follow-up for the 9 outcomes scales used in this study. The resulting "new" factors became a subject of mean value analysis, correlation analysis and interpretation.

Because various scales had different rates of data completeness, factor analysis was initially performed using only the subset of the TKAR cohort where values on all 9 scales were complete. Thereafter, various sensitivity analysis tests were done to investigate generalizability of the results to the entire TKAR cohort. In this regard, we investigated stability of the factorial structure, as well as mean values and correlation coefficients.

## Results

One hundred and eighty six consecutive patients undergoing TKAR had data collected for this study. The TKAR cohort had a mean age of 68.0 ± 10.8 (range 24.5 – 89.0), an equal representation of males and females, and was predominantly white (80%). There was no single dominant reason for failure of a knee replacement with the majority of the TKAs failing for multiple reasons. Although it is well known that individual outcomes can vary by mode of failure, it was necessary for scale development here to include here all patients, leaving it to future application of the scale to determine more subtle differences between different patient subgroups [13]. No deaths occurred during the period of follow up.

Eight scales out of nine, except the Mental Component Score of the SF-36 showed significant improvement of mean values between baseline and six months follow up for the cohort as a whole (Table 1). Moreover, patients improved the most on KSS-knee rating (1.64) and physician severity assessment (1.42) when normalized improvement values were compared. The least improvement was in the LEAS score (0.33). In addition, improvements on individual scales were either not correlated or significantly positively correlated (Table 2). The absence of negative significant correlations confirmed at least the face validity of the nine applied measures.

**Table 1:** Change in average scale scores from baseline to 6-month follow-up.

**Scale**	**N**	**Baseline**	**Follow-up**	**Change**^‡^	**Normalized Change#**
SF-36, PCS	172	31.2 ± 7.3	37.2 ± 9.4	6.2 ± 9.1*	0.68*
SF-36, MCS	172	48.4 ± 12.3	50.5 ± 11.4	1.5 ± 11.5	0.13
WOMAC, Pain	177	9.9 ± 4.5	5.6 ± 4.5	4.2 ± 4.8*	0.88*
WOMAC, Stiffness	181	4.1 ± 1.9	3.1 ± 2.0	1.0 ± 2.1*	0.48*
WOMAC, Diff. of Function	177	33.9 ± 14.2	21.9 ± 15.1	11.8 ± 14.4*	0.82*
KSS, Knee Rating	105	40.8 ± 18.0	76.4 ± 15.2	37.8 ± 23.0*	1.64*
KSS, Functional Assessment	160	40.4 ± 21.4	62.7 ± 25.4	23.1 ± 27.9*	0.83*
LEAS	172	7.6 ± 2.5	8.5 ± 2.6	0.9 ± 2.8*	0.33*
Physician Severity Assessment	142	6.8 ± 2.1	2.8 ± 2.1	4.1 ± 2.9*	1.42*

‡ Positive values denote improvement

# Normalized by standard deviation of change

* Change is significant at the 0.001 level (2-tailed)

**Table 2:** Correlation matrix of changes of scale scores.

	D1	D2	D3	D4	D5	D6	D7	D8	D9
D1	1.00	-.10	.26**	.27**	.28**	.47**	.54**	.27**	.13
N	172	172	132	161	170	167	168	146	93
D2		1.00	.17*	.21**	.03	.14	.20*	.17*	.28**
N		172	132	161	170	167	168	146	93
D3			1.00	.27**	-.03	.33**	.30**	.63**	.26*
N			142	133	138	135	136	128	84
D4				1.00	.02	.30**	.31**	.27**	.17
N				172	168	164	165	149	97
D5					1.00	.47**	.40**	.02	.10
N					181	176	175	155	100
D6						1.00	.71**	.26**	.32*
N						177	175	153	99
D7							1.00	.36**	.17
N							177	153	99
D8								1.00	.24*
N								160	101
D9									1.00
N									105

First number in every cell (D1-D9) specifies correlation coefficient, second number (N) – number of cases included into calculations.

D1-D9 indicate changes of scale scores from baseline to follow-up for SF-36 PCS (D1), SF-36 MCS (D2), Physician Severity Assessment (D3), LEAS (D4), WOMAC Stiffness (D5), WOMAC Pain (D6), WOMAC Difficulty of Functioning (D7), KSS Functional Assessment (D8), KSS Knee Rating (D9).

** Correlation is significant at the 0.01 level (2-tailed).

* Correlation is significant at the 0.05 level (2-tailed).

The 9 × 9 matrix demonstrated the highest correlations (> = 0.45) between improvements in SF-36 PCS and WOMAC pain scores, SF-36 PCS and WOMAC difficulty in function scores, physician assessment of patient severity and KSS function scores, WOMAC stiffness and WOMAC pain scores, WOMAC pain and WOMAC difficulty in function scores.

Sixty nine cases (the factor analysis cohort) had data completed for all nine scales and formed the basis for factor analysis. This technique elucidated four orthogonal factors that explained 73% of total variance, a high value compared to the constituent measurement scales (Table 3). Factor 1 was mainly composed of change in SF-36 PCS and the 3 WOMAC components. Factor 2 was mainly based on change in KSS- functional component, change in physician severity assessment and change in LEAS score. Factor 3 depended mainly on change in SF-36 MCS; factor 4 depended mainly on change in KSS-knee rating. Factor 1 and factor 2, composed of four and three original scales respectively, showed sufficient internal consistency based on Cronbach alpha (Factor 1 – 0.75; Factor 2 – 0.66).

**Table 3:** Rotated component matrix with four factors V1-V4.

	Factor			
	
	V1	V2	V3	V4
D7	**.72**	.46	.09	-.05
D6	**.78**	.32	-.02	.20
D5	**.77**	-.19	.16	-.02
D9	.04	.08	.12	**.96**
D8	.03	**.85**	-.15	.09
D3	.16	**.75**	.12	.26
D4	.15	**.62**	.21	-.14
D1	**.65**	.20	-.50	-.04
D2	.10	.17	**.91**	.13

D1-D9: changes of scale scores. Details see Table 4.

Bold font indicates loadings greater or equal than 0.6

Mean scores for improvement factors in this "factor analysis cohort" were positive and significantly different from zero (except for V3), indicating improvement (Table 4). The magnitude of such improvement (using normalized values) was highest in V4 (1.60), followed by V2 and V1 respectively. Moreover, correlations between the 4 factors were all positive but significant only between V1 and V2 (0.37, p < 0.01) and between V3 and V4 (0.24, p < 0.05).

**Table 4:** Average Scores of the Improvement Factors for Various Cohorts.

	"Factor Analysis" cohort			"Total" cohorts		
Factor	N	Mean ± SD	Normalized Mean#	N	Mean ± SD	Normalized Mean#

V1	69	0.82 ± 0.77*	1.07*	165	0.72 ± 0.79*	0.91*
V2	69	0.93 ± 0.75*	1.24*	120	0.88 ± 0.76*	1.16*
V3	69	0.21 ± 1.00	0.21	172	0.13 ± 1.00	0.13
V4	69	1.60 ± 1.00*	1.60*	105	1.64 ± 1.00*	1.64*

"Factor Analysis" cohort contains cases (69) when all four factors can be computed; Four "Total" cohorts contain cases (105–172) when at least one factor can be computed.

# Normalized by standard deviation

* Significantly different from 0 at the 0.01 level (2-tailed)

A noteworthy feature of this procedure is the stability in this factorial structure when the number of cases with complete data included into the analysis was increased by lowering the number of scales used. For example, when D9 (i.e. KSS knee rating, equivalent to Factor 4) was excluded from the analysis, 109 cases had complete data on all 8 scales left for analysis. In this analysis, however, the other factors again depended mainly on the same constituent scales. Similar findings were observed when other scales were sequentially excluded. The factorial structure was thus shown to be stable with varying combinations and sample sizes and therefore representative of the entire TKAR cohort.

The sensitivity of the factors' mean values was examined by comparing the "factor analysis cohort" calculations for each of the 4 factors with all available cases in the entire patient cohort where data was complete on relevant scales (Table 4). For example, Factor 1 (V1) was calculated from the original factor analysis cohort cases (N = 69) and then recalculated using all cases in the entire TKAR cohort with complete data on the constituent scales of Factor 1 (D1, D5, D6, and D7; N = 165). Mean values of V1 (as well as V2, V3, and V4) in the "factor analysis cohort" (0.82 ± 0.77) and the larger cohort (0.72 ± 0.79) were not significantly different from each other. In addition, normalized means of the recalculated factors (except V3) were positive and significantly different from zero, indicating improvement of the same order of magnitude as for the factor analysis cohort (Table 4).

Finally, changes in correlations between the four derived variables were studied. When factors were derived from a larger number of cases, the pattern of correlation was very similar to that of the original factor analysis cohort (Table 5). In fact, the highest correlation was observed between factors V1 and V2 (0.35, p < 0.01).

**Table 5:** Correlation Matrix of the Improvement Factors for Various Cohorts.

	V1	V2	V3	V4
V1	1.00 (69)	0.37 (69)**	0.07 (69)	0.08 (69)
	1.00 (165)	0.35 (109)**	0.10 (96)	0.24 (89)*
V2		1.00 (69)	0.21 (69)	0.19 (69)
		1.00 (120)	0.21 (113)*	0.21 (76)
V3			1.00 (69)	0.24 (69)*
			1.00 (172)	0.28 (93)**
V4				1.00 (69)
				1.00 (105)

First number in every cell – correlation coefficient; second number – the sample size. "Factor Analysis" cohort (first line in every cell) contains cases (69) when all four factors can be computed; Six other cohorts (second line in every cell) contain cases when at least two factors can be computed.

** Correlation is significant at the 0.01 level (2-tailed).

* Correlation is significant at the 0.05 level (2-tailed).

## Discussion

We have described a prospective clinical study that has afforded us the opportunity to evaluate the impact of TKAR on multiple health dimensions [14]. As a result we have been able to develop a new approach to measuring outcomes in TKA patients. It is based entirely on dynamic changes in patient factors over time (what we are terming 'improvement') and not the static cross sectional measurements obtained with individual scales. The scale also presents data from a large series of instruments in a simple economical fashion. Although the potential benefit of aggregated outcome measures has been recognized previously it has never been used in this clinical manner [15].

The current study demonstrates that improvement following TKAR has a multidimensional structure. By using combinations of quality of life measures, usually a global and a specific instrument, most current studies tacitly acknowledge this fact [2,3,16]. However, no previous study has analyzed the actual dimensional structure of improvement. It is not a new observation that, although accurate in what they are measuring, many studies are assessing aspects of improvement with no reflection on how these measurements interact as a whole and how, or even whether, they reflect all the dimensions of improvement [17]. It is noteworthy in this regard that although some of the highest correlations in the current study were observed between measures that were extracted from the same instrument, high correlations were also observed between measures extracted from different instruments. This leads us to believe that the different instruments used here are not measuring entirely independent dimensions of improvement. In order to streamline all of these issues, our ultimate objective is to arrive at a single measure that will address whole patient improvement.

Factor one largely reflects the patient's perception of relative improvement in physical capabilities. It is mainly composed of changes in the physical component score of the SF-36, a general health measure, and changes in all components of the WOMAC, a disease specific measure. The second factor is based on changes in the KSS functional component, physician based VAS of patient severity and on changes in the LEAS. Factor 2 thus reflects the actual activity level of the patient. The presence of the physician's assessment of the severity of the patient's condition here is of interest and leads us to hypothesize if not conclude that this assessment, and thus the physician perceived urgency of need for TKAR, is based largely on the physician's assessment of the individual patient's activity or lack thereof.

Factor 3 reflects the mental status of the patient, being composed of the Mental Component Score of the SF-36. Describing the MCS as an independent element contributing to the measurement of improvement may at first appear counter-intuitive when it is recalled that the mean MCS did not change at all between the baseline and six month follow-up time points. What this does indicate though is that there must be significant changes taking place for individual patients that correlate with improvement in an independent way from the other scales which is not apparent when the population mean score change is calculated. The final Factor was found to signify the objective clinical status of the knee being composed mainly of changes in Knee Society Score clinical assessments. In summary, therefore, we can conclude that the 4 main aspects of improvement we measure with the current instruments in common use are patient and physician perceived general and specific functionality, actual patient activity, mental status and objective clinical assessment.

Although there exist more than the 9 conventional scales from different instruments used here for assessing TKA outcomes, those used here are all in common use and reflect, in category if not in every detail, the other available tests and the many possible subjective and objective measures they encompass [6,18-20]. As mentioned above most studies of arthroplasty outcomes use a global health score and a disease specific scale in order to try to achieve a more representative description of outcomes. The findings of the factor analysis performed here indicate that this 2 dimensional approach can be further refined and that improvement following TKAR is actually composed of 4 independent factors.

Despite these findings, however, it is entirely possible that there may be other instruments or methods of assessment not specifically assessed here that can add further depth or accuracy to the factors we have described. We have essentially established the actual *dimensionality *of improvement and further prospective application will further test the stability of this structure. In such future prospective work, elements of other instruments can thus be tested as exploratory data. Analysis will then ultimately determine the constituents of the definitive instrument which, we hypothesize, will be based on the 4 factors. For example, a new instrument focused on these 4 factors could comprise a panel of a relatively small number of very focused or specific questions as opposed to the very large number of questions required in the quite cumbersome combination of other instruments typically required for modern clinical studies.

We have therefore developed a paradigm, with the four factors described here giving the structure on which the new multi-dimensional tool we feel is necessary in this area will be developed. We have shown that each of the 4 dimensions or factors found here require separate representation and analysis in order to accurately measure improvement in our total knee arthroplasty patients. In fact, this 4 factor paradigm was more successful in measuring variability (73% versus 30%) when compared to a recent NIH report that reported on the use of the standard measurement techniques [1].

Finally, an important feature of this new scale is its potential importance in predicting outcomes. Further prospective work with the scale will determine its place in this regard but we anticipate that the Improvement Scale will eventually give us the critical information regarding which dimensions of improvement are relatively most important in certain patient populations and what dimensions most accurately determine outcomes for these patient groups. It should better enable us to decide on the most appropriate nature and timing of intervention based on a comprehensive understanding of overall patient improvement, thus maximizing patient outcomes.

## Competing interests

The author(s) declare that they have no competing interests.

## Authors' contributions

HG, BB, KJS and KJM all made substantial contributions to conception and design, acquisition of data, analysis and interpretation of data; KJM, KJS and HG were involved in drafting the manuscript or revising it critically for important intellectual content; and all authors have given final approval of the version to be published.
